# Intermittent Fasting and Sleep: A Review of Human Trials

**DOI:** 10.3390/nu13103489

**Published:** 2021-10-01

**Authors:** Mara McStay, Kelsey Gabel, Sofia Cienfuegos, Mark Ezpeleta, Shuhao Lin, Krista A. Varady

**Affiliations:** Department of Kinesiology and Nutrition, University of Illinois at Chicago, Chicago, IL 60045, USA; mmcsta2@uic.edu (M.M.); kdipma2@uic.edu (K.G.); scienf2@uic.edu (S.C.); mezpel2@uic.edu (M.E.); slin89@uic.edu (S.L.)

**Keywords:** intermittent fasting, time restricted eating, alternate day fasting, sleep quality, sleep duration, sleep apnea, insomnia, obesity

## Abstract

This review examines the effects of two popular intermittent fasting regimens on sleep in adults with overweight and obesity. Specifically, the effects of time restricted eating (TRE; eating all food within a 4–10 h window) and alternate day fasting (ADF; 600 kcal fast day alternated with ad libitum feast day) on sleep quality, sleep duration, sleep latency, sleep efficiency, insomnia severity, and risk of obstructive sleep apnea, will be summarized. The role of weight loss will also be discussed. Results from our review reveal that the majority of these trials produced weight loss in the range of 1–6% from baseline. Sleep quality and sleep duration remained unaltered with TRE and ADF, as assessed by the Pittsburgh Sleep Quality Index (PSQI). The effects of intermittent fasting on sleep latency and sleep efficiency are mixed, with one study showing worsening of these parameters, and others showing no effect. Insomnia severity and the risk of obstructive sleep apnea remained unchanged in the trials assessing these metrics. Taken together, these preliminary findings suggest that TRE and ADF produce mild to moderate weight loss (1–6%) but their effects on sleep remain unclear. Solid conclusions are difficult to establish since participants in the studies had healthy sleep durations and no clinical insomnia at baseline, leaving little room for improvement in these metrics. Moreover, none of the trials were adequately powered to detect statistically significant changes in any measure of sleep. Future well-powered trials, conducted in individuals with diagnosed sleep disturbances, will be necessary to elucidate the effect of these popular diets on sleep.

## 1. Introduction

The prevalence of overweight and obesity continues to increase, currently afflicting more than 72% of Americans [1]. Obesity is associated with reduced sleep duration, i.e., <7 h of sleep per night, and poor sleep quality [2,3,4]. Those with overweight and obesity are also more likely to report insomnia, increased sleep disturbances, and daytime sleepiness [5,6,7,8]. 

Intermittent fasting has emerged as a promising dietary intervention for weight loss in recent years. Two of the most studied fasting regimens include time restricted eating (TRE) and alternate day fasting (ADF). TRE requires individuals to eat within a certain window of time each day (typically 4–10 h) and then fast for the remaining hours of the day. ADF, on the other hand, involves a “fast day” where one consumes 0–600 kcal/d, alternated with a “feast day” where one is permitted to eat ad libitum. TRE produces mild to moderate weight loss of 1–4% in adults with overweight and obesity over 1–16 weeks [9,10,11]. ADF, on the other hand, generally results in slightly greater weight loss (3–7%) over a similar time frame, when compared to TRE [12,13,14,15]. 

In recent years, it has been speculated that intermittent fasting may improve sleep [16]. One of the postulated mechanisms involves improving circadian rhythmicity. Intermittent fasting may strengthen the peripheral circadian rhythm via limiting food intake during the evening and nighttime. This can, in turn, restore the homeostatic nature of the internal clock, which may have beneficial effects on sleep in those with erratic sleep patterns. Another mechanism by which intermittent fasting may improve sleep is by reducing body weight. Weight loss has been shown to improve several sleep parameters, including sleep quality, sleep duration, and the risk for obstructive sleep apnea [8,17,18,19,20]. The effects of intermittent fasting (TRE and ADF) on sleep have been investigated in a handful of trials [21,22,23,24,25,26,27,28,29]. The goal of this review is to summarize the effects of TRE and ADF on various sleep parameters, including sleep quality, sleep duration, sleep latency, sleep efficiency, insomnia severity, and risk of obstructive sleep apnea, in adults with overweight and obesity. The degree of weight loss required to produce improvements in these sleep variables will also be discussed.

## 2. Methods—Human Trial Selection

A Pubmed search was conducted using the following key words: “sleep”, “sleep quality”, “sleep duration”, “sleep latency”, “sleep efficiency”, “insomnia”, “sleep apnea”, “weight loss”, “body weight”, “intermittent fasting”, “fasting”, “meal timing”, “meal frequency”, “intermittent energy restriction”, “alternate day fasting”, “5:2 diet”, “time restricted eating”, “time restricted feeding”, “clinical trial”, “human.” Inclusion criteria for research articles were as follows: (1) randomized controlled trials and non-randomized trials, (2) adult male and female participants, and (3) endpoints that included changes in body weight and sleep metrics. The following exclusion criteria were applied: (1) cohort and observations studies, (2) fasting performed as a religious practice (Ramadan or Seventh Day Adventist), and (3) trial durations of less than 1 week. A total of 203 studies were identified. Out of these, after examination of title (*n* = 156), abstracts (*n* = 22), and full-texts (*n* = 16), 9 studies were finally included in the review. Our search retrieved 8 human trials of TRE and one human trial of ADF (Table 1). We were not able to find any studies of the 5:2 diet (i.e., two 600 kcal fast days, two times per week) that examined sleep parameters, so this popular form of fasting is not included in this review.

## 3. Effect of Intermittent Fasting on Body Weight 

Accumulating evidence suggests that weight loss may improve sleep [8,17,18,19,20]. In a recent weight loss trial, successful weight loss maintainers reported better sleep quality and longer sleep durations versus individuals with obesity who did not lose weight [30]. Weight loss is also associated with a reduced risk of obstructive sleep apnea [20,31]. Obesity contributes to sleep apnea via narrowing of the upper airway and reducing functional residual capacity. Weight loss has been shown to alleviate these physiological dysfunctions and decrease the severity of sleep apnea [31,32].

The weight loss threshold at which participants experience significant sleep improvements is not fully understood. Weight loss greater than 5% relative to baseline is considered clinically significant due to meaningful improvements in traditional cardiometabolic risk factors, including plasma lipid levels, blood glucose, and blood pressure [33]. Recent weight loss studies have demonstrated improvements in sleep quality and duration with >5% weight loss [8,17,18]. In contrast, risk of obstructive sleep apnea is improved when participants with obesity achieve 10% weight loss from baseline [34]. Thus, it stands to reason that body weight reductions exceeding 5% may be needed to improve sleep quality and duration, while weight reductions exceeding 10% may be required to improve sleep apnea severity. 

The effect of TRE on weight loss and sleep was evaluated in eight trials (Table 1). In these trials, participants were permitted to eat ad libitum within a 4-h, 6-h, 8-h, 9-h, or 10-h window. Limiting the eating window to these time frames produced 20–30% reductions in energy intake without calorie counting [21,22,23,24,25,26,27,28]. Due to these reductions in energy intake, subjects lost 1–4% of body weight over 1–16 weeks [21,22,23,24,25,26,27,28]. Interestingly, shorter eating windows did not correspond to greater weight loss. Trial duration, in contrast, played a role in determining the degree of weight loss achieved. Specifically, weight loss was greater during the 8–16 week trials (2–4% weight loss) compared to the 1–4 week trials (0–1% weight loss). It is important to note that none of the TRE trials reviewed here produced clinically significant weight loss (>5% from baseline). This should be taken into consideration when interpreting the reported sleep data. 

Only one ADF trial has evaluated changes in body weight and sleep. In this trial by Kalam et al. [29], subjects consumed 600 kcal on the fast day, and ate ad libitum on alternating feast days. The trial was divided into a 12-week weight loss period followed by a 12-week weight maintenance period. The ADF regimen was combined with a low carbohydrate/high protein background diet (30% carbohydrates, 35% protein, and 35% fat). Subjects consumed shakes throughout the trial on the fast days and feast days to assist in meeting their carbohydrate and protein goals. Body weight decreased by 6% at the end of the 12-week weight loss period, and this degree of weight loss was sustained during the 12-week maintenance period. Thus, the ADF low carbohydrate/high protein regimen was effective in producing clinically significant weight loss (>5%) in men and women with obesity.

## 4. Effects of Intermittent Fasting on Sleep Parameters

### 4.1. Sleep Quality

Sleep quality was measured in five of eight TRE studies using the validated PSQI questionnaire [22,23,24,26,27]. This 19-question self-report assesses sleep quality over the past month [35]. The PSQI survey generates a score ranging from 0–21. A total score greater than 5 indicates poor sleep quality. In three TRE studies [22,23,27], subjects scored higher than 5 on the PSQI questionnaire at baseline, indicating that they were “poor sleepers” at the onset of treatment. In the other two TRE studies, [24,26] subjects scored below 5 on the PSQI survey, indicating that they were “good sleepers” at baseline. Results reveal that TRE had no effect on sleep quality in either “poor sleepers” [22,23,27] or “good sleepers” [24,26]. One explanation for this lack of effect could be the minimal amount of weight loss achieved. Participants in these five TRE studies lost 2–3% of body weight over 4–12 weeks [22,23,24,26,27]. It is possible that favorable changes in sleep quality may only be achieved with clinically significant weight reductions (>5%) [8,17,18]. 

Interestingly, other survey tools revealed improvements in sleep quality during TRE. In addition to using PSQI, Wilkinson et al. [27] measured sleep quality with the smartphone app, myCircadianClock (mCC), during 12 weeks of 10-h TRE. Each morning, participants rated the previous night’s sleep in the mCC app. By week 12, subjects reported a 23% increase in nightly restful sleep relative to baseline [27]. Kestyus et al. [25] also used an alternative survey tool to measure sleep quality. In this trial, subjects rated their perceived sleep quality using a visual analogue scale (VAS), which ranged from 0 (worst possible sleep quality) to 100 (best possible sleep quality). After 12 weeks of 9-h TRE, sleep quality improved significantly by 10 points (from 65 to 75) on the VAS [25]. The reason the findings generated from the mCC app and the VAS differ from that of the PSQI remains unclear. However, it is important to note that the mCC and VAS tools measured sleep quality on a daily basis. These tools allowed for frequent, repeated measurements, reducing recall bias and potentially leading to more accurate real-time assessments of sleep quality. 

The effect of ADF on sleep quality was also measured. Kalam et al. [29] administered the PSQI survey to subjects with obesity at baseline, at week 12 (end of the weight loss period), and at week 24 (end of the weight maintenance period). Participants experienced no significant change in sleep quality throughout the trial. 

Kalam et al. [29] also performed a sub-analysis to examine the effects of the ADF intervention in “poor sleepers” (PSQI score >5) vs. “good sleepers” (PSQI score <5). The “poor sleepers” demonstrated a significant improvement in sleep quality at week 12, though the effect was washed out during the maintenance period by week 24. This finding was surprising given that the average weight reduction of 6% achieved during the weight loss period was sustained during the maintenance period. In “good sleepers”, mean PSQI was not significantly different at week 12 or 24, compared to baseline. It is worth noting that this trial evaluated sleep quality as a secondary outcome measure. Thus, the trial was most likely not adequately powered to detect significant changes in sleep quality.

It is possible that initial sleep improvements in “poor sleepers” were partially mediated by changes in macronutrient composition of the diet. Participants in this ADF trial consumed a high-protein (35% of energy) background diet [29]. Recent studies have reported reduced wake episodes and improved global sleep quality when participants followed a high-protein (25–55% of energy) diet [36,37,38]. Why the improvements in sleep quality did not persist past the weight loss period, despite continuing the high protein diet, is unclear.

### 4.2. Sleep Duration

Experts recommend that adults sleep 7 to 9 h per night [39]. However, only 65% of American adults regularly meet this recommendation [40]. Sleep duration was measured in all eight TRE studies using various methods, including the Pittsburgh Sleep Quality Index (PSQI) [22,23,24], self-report diary [25], subjective self-assessment survey [28], and accelerometers [21,26,27]. Seven of the eight TRE trials reported no change in sleep duration [21,22,23,24,25,26,27]. However, it is important to note that the majority of subjects in these studies were already sleeping at least 7 h per night at baseline [21,23,24,25,27]. As such, it is not surprising that sleep duration did not change in healthy sleepers by the end of the intervention. It will be of interest for future studies to examine whether TRE can improve sleep duration in subjects who sleep less than 7 h per night at the onset of treatment. 

The only study to report changes in sleep duration was the 10-h TRE trial by Gill & Panda [28]. Participants completed a subjective self-assessment survey and answered the question “Do you feel you are getting enough sleep?” on a scale of 1 to 10, at baseline and post-treatment. Study subjects reported a 1.5 point improvement in sleep duration at the end of the 16-week trial, relative to baseline [28]. However, this study is flawed in that it did not use a validated survey tool to measure sleep duration. The study also failed to report the number of minutes that sleep duration increased. These limitations should be taken into consideration when interpreting the data.

The effect of ADF on sleep duration was measured by Kalam et al. [29]. After 24 weeks of an ADF-low carbohydrate/high protein diet, subjects achieved 6% weight loss, but sleep duration remained unchanged [29]. However, subjects in this study were already sleeping at least 7 h per night at baseline. This could explain why no improvement was observed.

### 4.3. Sleep Latency & Sleep Efficiency 

Sleep latency refers to the amount of time it takes to fall asleep, whereas sleep efficiency measures the percentage of time spent asleep while in bed [41,42]. The effect of TRE on sleep latency and efficiency was evaluated in four trials [22,23,26,27]. In the 8-h TRE study by Lowe et al. [26] subjects with overweight and obesity were provided Oura ring sleep trackers to wear daily over 12 weeks. Sleep latency and efficiency scores worsened in the TRE groups versus controls by the end of the study. Interestingly, the Lowe et al. study [26] noted a significant decrease in the TRE group’s daily movement over the trial, as measured by the Oura ring. Physical activity has been shown to improve both sleep latency and efficiency [43,44]. Thus, it is likely that the decrease in daily movement may have negatively impacted participant sleep latency and efficiency scores.

Similarly, Wilkinson et al. [27] provided patients a wrist-actigraphy device to objectively track sleep efficiency. No change in sleep efficiency was observed during 12 weeks of 10-h TRE [27]. Cienfuegos et al. [23] and Parr et al. [22] derived sleep latency and sleep efficiency scores from the PSQI survey. Cienfuegos et al. [23] found no change in sleep latency in the 4-h and 6-h TRE groups by the end of the 8-week trial. Likewise, Parr et al. [22] observed no effect on sleep latency nor sleep efficiency after 4 weeks of 9-h TRE, in subjects with overweight and obesity. Daily activity counts remained unchanged in the Wilkinson et al. [27] and Cienfuegos et al. [23] trials, which may have contributed to the lack of effect on sleep efficiency and sleep latency metrics [43,44]. 

### 4.4. Insomnia Severity

Insomnia is defined as a difficulty in initiating or maintaining sleep [45]. It has been postulated that TRE diets, which involve fasting for 2–5 h before bedtime, may lower rates of insomnia. Specifically, abstaining from consuming fatty and acidic food before bed may decrease nighttime heartburn and acid reflux, which could contribute to lower rates of insomnia [46,47].

The effects of 4–6-h and 8-h TRE on insomnia severity were evaluated in two trials using the validated Insomnia Severity Index (ISI) [23,24]. The ISI is a 7-item questionnaire used to estimate insomnia severity over the previous week [48]. Each item is rated on a 5-point Likert scale (where 0 indicates no problem, and 4 indicates a very severe problem), yielding a total score of 0–28. Total ISI scores are interpreted as follows: no clinically significant insomnia (0–7), sub-threshold insomnia (8–14), moderate severity insomnia (15–21), or severe insomnia (22–28). Participants in both the Cienfuegos et al. [23] and Gabel et al. [24] trials demonstrated no clinically significant insomnia at baseline. After 8–12 weeks of TRE, no changes in insomnia severity were observed in either trial. Given the lack of insomnia at baseline, it is not surprising that insomnia severity remained unaltered throughout these intervention periods. The lack of clinically significant weight loss may have also precluded these trials from observing a significant change in insomnia severity.

The effect of ADF on insomnia severity was evaluated in the trial by Kalam et al. [29]. After 24 weeks of ADF combined with a low carbohydrate/high protein diet, insomnia severity remained unchanged in men and women with obesity. The authors also performed a sub-analysis to evaluate whether “poor sleepers” responded differently to ADF versus “good sleepers.” The baseline ISI score in “poor sleepers” was 12, indicating sub-threshold insomnia. By week 12, “poor sleepers” lost 5% body weight, and the ISI score was significantly reduced to 8. By week 24, the 5% weight loss was maintained, but the ISI score increased to 9, which was no longer significant compared to baseline. The reason why this occurred is unclear, as recent trials have demonstrated that sustained weight maintenance is associated with lasting improvements in insomnia severity [4]. On the other hand, the group of “good sleepers” achieved 6% weight loss but displayed no insomnia at baseline. Not surprisingly, ISI scores in the “good sleeper” cohort did not change at any point during the study. 

### 4.5. Risk of Obstructive Sleep Apnea

Obstructive sleep apnea refers to the frequent collapse of the upper airway during sleep, resulting in obstruction of normal breathing [32]. Obstructive sleep apnea has been linked to an increased risk of metabolic syndrome, cardiovascular disease, and type 2 diabetes [49]. Overweight and obesity is considered the most significant risk factor for sleep apnea, and weight loss is highly associated with improvement in sleep apnea [20]. 

Only one TRE trial [23] has evaluated changes in the risk for obstructive sleep apnea in participants with obesity. Changes in this sleep metric were quantified by the Berlin Questionnaire [50]. In the trial by Cienfuegos et al. [23], a high risk of sleep apnea was present in 44% of 4-h TRE participants and 47% of 6-h TRE participants at baseline. At the end of the 8-week study, sleep apnea risk decreased substantially, to 25% in the 4-h TRE cohort and 20% in the 6-h TRE group. Interestingly, this numerical reduction was not significantly different when post-treatment values were compared to baseline. However, the trial was likely underpowered to detect significant changes in risk for sleep apnea, since sleep metrics were not accounted for in the original power calculation [23]. Moreover, weight loss was minimal (3%) in both the 4-h TRE and 6-h TRE groups, which may have played a role in the lack of effect noted. 

The effect of ADF on obstructive sleep apnea risk was evaluated in the study by Kalam et al. [29]. At baseline, 45% of participants were at high risk for sleep apnea. After 24 weeks, no statistically significant decrease in the risk of sleep apnea was observed, despite the 6% weight loss achieved by these subjects. A previous longitudinal, population-based trial showed that 10% weight loss was required to produce a 26% decrease in apnea severity [34]. In view of these previous findings, it is possible that the study by Kalam et al. [29] did not produce enough weight loss to observe improvements in sleep apnea risk in these subjects with obesity.

## 5. Summary of Findings

Taken together, these findings suggest that TRE and ADF have little effect on sleep metrics. Sleep quality remained unchanged with TRE and ADF, when assessed via the PSQI survey [22,23,24,26,27,29]. However, two TRE trials [25,27] showed sleep quality improvements when surveying participants using daily self-report methods (mCC app and VAS scales). Sleep duration also remained unchanged in the majority of the TRE and ADF trials reviewed here [21,22,23,24,25,26,27,29]. Though it should be mentioned that most subjects in these trials were already getting sufficient sleep at baseline (>7 h per night), thus, this lack of effect is not surprising. The effect of TRE on sleep latency and sleep efficiency is mixed. In the study by Lowe et al. [26] participants experienced worsened sleep latency and sleep efficiency scores as measured by the Oura ring device. Conversely, three other studies revealed no effect on sleep latency and efficiency when assessed by wrist actigraphy and the PSQI survey [22,23,27]. As for insomnia severity, no significant change was observed with fasting, though it should be noted that subjects in these trials did not have insomnia at baseline [23,24]. Sleep apnea risk did not change in the TRE and ADF trials that evaluated this metric [23,29]. 

While preliminary findings may suggest that TRE and ADF have little effect on sleep, it is important to note that these fasting protocols did not have a *detrimental* impact on sleep. It is likely that the degree of weight loss achieved in these trials was, for the most part, not sufficient to produce significant improvements in sleep metrics. As mentioned previously, 5–10% weight loss may be required to improve these parameters [8,17,18]. Since the TRE protocols only produced 1–4%, this may explain why these sleep variables remained vastly unaltered. It remains unclear why the ADF study, which produced 6% weight loss, did not change any of the sleep metrics. 

## 6. Directions for Future Research

Future intermittent fasting studies should examine if greater degrees of weight loss (5–10% from baseline) during TRE and ADF produce significant changes in sleep. It will also be of interest to examine whether fasting can improve sleep in individuals with severe insomnia, sleep apnea, or persistent sleep disturbances. Prospective research should prioritize using wrist actigraphy as these devices provide the most robust assessments of sleep duration and sleep efficiency [51,52]. Future trials may also want to focus on standardization of the participant eating window. Specifically, it will be of interest to see if the placement of the TRE eating window, namely earlier or later in the day, affects sleep. Recent research suggests that early time restricted eating (eating all food before 3 p.m.) is linked to enhanced circadian gene clock expression [53,54]. In view of this, it is possible that certain TRE protocols may help to properly align circadian rhythms in a way that promotes improved sleep [55]. 

## 7. Limitations to the Current Body of Evidence

These preliminary studies have several limitations that should be considered when interpreting this body of evidence. First, the trials all had small sample sizes (*n* = 8–116) and measured sleep as a secondary exploratory outcome measure. Thus, it is highly likely that none of these trials were adequately powered to detect statistically significant changes in any measure of sleep. Second, these trials were conducted primarily in healthy sleepers who did not have sleep disturbances at baseline. Third, some of the tools implemented in these trials lacked robust validation, i.e., mCC app and VAS scales for sleep quality. Fourth, caffeine intake was not monitored during these trials. As such, participants may have consumed caffeine late into the evening, which could have affected their sleep quality and duration. Fifth, subject chronotype was not assessed. Chronotype can be easily measured by the morningness-eveningness questionnaire [56]. The evening chronotype is associated with consuming larger meals later in the day and higher rates of sleep apnea in adults with obesity [57]. As such, it will be important to examine how the sleep habits of individuals of varying chronotypes respond to intermittent fasting.

## 8. Conclusions

In conclusion, these preliminary findings suggest that TRE and ADF produce mild to moderate weight loss (1–6%) but their effects on sleep remain unclear. Solid conclusions are difficult to establish since participants in the studies had healthy sleep durations and no clinical insomnia at baseline, leaving little room for improvement in these metrics. Moreover, none of the trials were adequately powered to detect statistically significant changes in any measure of sleep. Thus, the impact of fasting on sleep quality, sleep duration, sleep latency, sleep efficiency, insomnia severity, or risk of obstructive sleep apnea still remain largely unknown. Nevertheless, these pilot findings may provide valuable data to design and formulate future well-powered studies. Longer-term trials with large sample sizes will undoubtedly be necessary to fully elucidate the effect of these popular diets on sleep.

## Figures and Tables

**Table 1:** Effect of intermittent fasting on sleep parameters in adults with overweight and obesity.

Reference	Subjects	Diet Length	Design and Intervention Groups	Weight Loss (% Change)	Dietary Intake (Change)	Sleep Quality (Change)	Sleep Duration (Change)	Sleep Latency (Change)	Sleep Efficiency (Change)	Insomnia Severity (Change)	Sleep Apnea Risk (Change)
**Time restricted eating**											
Hutchison et al., 2019 [21]	*n* = 15, M Overweight Obese Prediabetic	1 week	**RT: Crossover** 1. 9-h TRE (8 a.m.–5 p.m.) 2. 9-h TRE (12–9 p.m.)	1. ↓ 1% * 2. ↓ 1% *	-	-	1. ∅ (Accelerometer) 2. ∅ (Accelerometer)	-	-	-	-
Parr et al., 2020 [22]	*n* = 19, MF Overweight Obese Type 2 Diabetes	4 weeks	**Single-arm** 1. 9-h TRE (10 a.m.–7 p.m.)	1. ∅	1. ∅ kcal %P: ∅, %F: ∅, %C: ∅	1. ∅ (PSQI)	1. ∅ (PSQI)	1. ∅ (PSQI)	1. ∅ (PSQI)	-	-
Cienfuegos et al., 2021 [23]	*n* = 58, MF Obese No Diabetes	8 weeks	**RCT: Parallel-arm** 1. 4-h TRE (3–7 p.m.) 2. 6-h TRE (1–7 p.m.) 3. Control (no meal timing restrictions)	1. ↓ 3% *^,†^ 2. ↓ 3% *^,†^ 3. ∅	1. ↓ 530 kcal *^,†^ %P: ∅, %F: ∅, %C: ∅ 2. ↓ 570 kcal *^,†^ %P: ∅, %F: ∅, %C: ∅ 3. ∅ kcal %P: ∅, %F: ∅, %C: ∅	1. ∅ (PSQI) 2. ∅ (PSQI) 3. ∅ (PSQI)	1. ∅ (PSQI) 2. ∅ (PSQI) 3. ∅ (PSQI)	1. ∅ (PSQI) 2. ∅ (PSQI) 3. ∅ (PSQI)	-	1. ∅ (ISI) 2. ∅ (ISI) 3. ∅ (ISI)	1. ∅ (Berlin) 2. ∅ (Berlin) 3. ∅ (Berlin)
Gabel et al., 2019 [24]	*n* = 23, MF Obese No Diabetes	12 weeks	**Single-arm** 1. 8-h TRE (10 a.m.–6 p.m.)	1. ↓ 3% ^†^	1. ↓ 341 kcal * %P: ∅, %F: ∅, %C: ∅	1. ∅ (PSQI)	1. ∅ (PSQI)	-	-	1. ∅ (ISI)	-
Kesztyus et al., 2020 [25]	*n* = 99, MF Overweight Obese No Diabetes	12 weeks	**Single-arm** 1. 8–9-h TRE (self-selected)	1. ↓ 2% *	-	1. ↑ 10 pts * (VAS survey)	1. ∅ (Self-report diary)	-	-	-	-
Lowe et al., 2020 [26]	*n* = 116, MF Overweight Obese No diabetes	12 weeks	**RCT: Parallel-arm** 1. 8-h TRE (12–8 p.m.) 2. Control (3 meals + snacks each day)	1. ↓ 2% * 2. ∅	-	1. ∅ (PSQI) 2. ∅ (PSQI)	1. ∅ (Oura ring) 2. ∅ (Oura ring)	1. ↓ 2.9 pts *^,†^ (Oura ring) 2. ∅ (Oura ring)	1. ↓ 5.2 pts *^,†^ (Oura ring) 2. ∅ (Oura ring)	-	
Wilkinson et al., 2020 [27]	*n* = 19, MF Overweight Obese Prediabetes	12 weeks	**Single-arm** 1. 10-h TRE (self-selected)	1. ↓ 3% *	1. ↓ 198 kcal * %P: -, %F: -, %C: -	1. ∅ (PSQI) ↑ 23% * (mCC phone app)	1. ∅ (Actigraphy)	-	1. ∅ (Actigraphy)	-	-
Gill & Panda, 2015 [28]	*n* = 8, MF Overweight Obesity No Diabetes	16 weeks	**Single-arm** 1. 10-h TRE (self-selected)	1. ↓ 4% *	-	-	1. ↑ 1.5 pts * (Self-assessment survey)	-	-	-	-
**Alternate day fasting**											
Kalam et al., 2021 [29]	*n* = 31, MF Obese No diabetes	24 weeks	**Single-arm** 1. ADF Fast day (600 kcal), Feast day (ad libitum) + low-carb/high protein diet	1. ↓ 6% *	1. ↓ 680 kcal * %P: ↑ 15% *, %F: ∅, %C: ↓ 14% *	1. ∅ (PSQI)	1. ∅ (PSQI)	-	-	1. ∅ (ISI)	1. ∅ (Berlin)

-: Not measured. ∅: Non-significant change. * *p* < 0.05, Significantly different from baseline (within group effect). ^†^ *p* < 0.05, Significantly different from the control or comparison group (between group effect). Abbreviations: ADF: Alternate day fasting, %C: Percent energy from carbohydrates, %F: Percent energy from fat, F: Female, ISI: Insomnia Severity Index, M: Male, %P: Percent energy from protein, PSQI: Pittsburgh Sleep Quality Index, RT: Randomized trial, RCT: Randomized controlled trial, TRE: Time restricted eating (prescribed eating window shown in parentheses), ↑: increase, ↓: decrease.
